# Draft Genome Sequence of Methyloferula stellata AR4, an Obligate Methanotroph Possessing Only a Soluble Methane Monooxygenase

**DOI:** 10.1128/genomeA.01555-14

**Published:** 2015-03-05

**Authors:** Svetlana N. Dedysh, Daniil G. Naumoff, Alexey V. Vorobev, Nikos Kyrpides, Tanja Woyke, Nicole Shapiro, Andrew T. Crombie, J. Colin Murrell, Marina G. Kalyuzhnaya, Angela V. Smirnova, Peter F. Dunfield

**Affiliations:** a S.N. Winogradsky Institute of Microbiology, Moscow, Russia; b Department of Marine Sciences, University of Georgia, Athens, Georgia, USA; c Los Alamos National Laboratory, Joint Genome Institute, Biosciences Division Genome Science B6, Los Alamos, New Mexico, USA; d School of Environmental Sciences, University of East Anglia, Norwich Research Park, Norwich, United Kingdom; e Department of Microbiology, University of Washington, Seattle, Washington, USA; f Department of Biological Sciences, University of Calgary, Calgary, Alberta, Canada

## Abstract

Methyloferula stellata AR4 is an aerobic acidophilic methanotroph, which, in contrast to most known methanotrophs but similar to Methylocella spp., possesses only a soluble methane monooxygenase. However, it differs from Methylocella spp. by its inability to grow on multicarbon substrates. Here, we report the draft genome sequence of this bacterium.

## GENOME ANNOUNCEMENT

Methyloferula stellata AR4 is a methanotroph of the *Alphaproteobacteria* family *Beijerinckiaceae*. It is a typical inhabitant of acidic wetlands and soils (1). It represents only the second known genus of methanotrophs (after Methylocella) lacking a particulate methane monooxygenase (pMMO) and an extensive intracytoplasmic membrane system. *Methyloferula* spp. and Methylocella spp. possess only a soluble methane monooxygenase (sMMO) and share some phenotypic characteristics, such as tolerance to low pH and the ability to fix dinitrogen. However, they differ with regard to their substrate utilization patterns. Methylocella species are facultative methanotrophs, which, in addition to C_1_ compounds, utilize acetate and several other organic acids, ethanol, and some short-chain alkanes (2–4). In contrast, M. stellata grows only on methane and methanol (1).

The draft genome sequence was generated at the Department of Energy (DOE) Joint Genome Institute (JGI) using Illumina technology (5). The combination of short-insert (insert size, ~250 bp) and long-insert (~9,500 bp) paired-end libraries produced 3,088 Mb of data (see http://www.jgi.doe.gov/). These were assembled with AllPaths version r41554 and computationally shredded into 10-kb overlapping fake reads (6). The Illumina data were also assembled with Velvet version 1.1.05 (7), computationally shredded into 1.5-kb overlapping fake reads, reassembled with Velvet, and shredded into 1.5-kb overlapping fake reads. The fake reads from the AllPaths and two Velvet assemblies, as well as a subset of the Illumina cross-linking and immunoprecipitation sequencing (CLIP) paired-end reads, were assembled using parallel Phrap version 4.24 (High Performance Software, LLC). Possible misassemblies were corrected with manual editing in Consed (8–10). The total estimated size of the M. stellata AR4 genome is 4.24 Mb (coverage, 735×), with an average G+C content of 59.5%. A single rRNA operon, 46 tRNAs, and 3,967 predicted protein-coding genes were identified.

The absence of pMMO-encoding genes and the presence of an operon encoding sMMO (*mmoXYBZDC*) are unique features shared only by *Methyloferula stellata* and Methylocella species (11). An additional soluble diiron monooxygenase, i.e., propane monooxygenase, which is present in Methylocella silvestris BL2 (3, 11), is lacking in M. stellata. Strain AR4 contains a large array of genes encoding various alcohol dehydrogenase quinoproteins, i.e., one MxaFI- methanol dehydrogenase (MDH), five XoxF-MDHs, and one alcohol dehydrogenase type 6a quinoprotein (12). Genes involved in tetrahydromethanopterin-linked C_1_ transfer and formate oxidation were also identified. Similarly to other *Beijerinckiaceae* methanotrophs (13), the genome of M. stellata contains the complete set of genes for the function of the Calvin-Benson-Bassham cycle and the serine pathway for carbon assimilation, as well as genes encoding enzymes of the tricarboxylic acid cycle. It lacks the ethylmalonyl-coenzyme A (CoA) pathway for glyoxylate regeneration but possesses a glyoxylate bypass. The number of membrane transporters in *Methyloferula stellata* AR4 is nearly the same as in Methylocella silvestris BL2 (13); M. silvestris BL2 however, possesses an acetate/glycolate transporter that is lacking in strain AR4. The genes involved in N_2_ fixation are organized as in Beijerinckia indica (14) into two genomic islands, with one additional *nifK* gene homologue located outside these islands.

### Nucleotide sequence accession number.

The M. stellata AR4 genome sequence was deposited in GenBank/EMBL under the accession no. ARWA00000000.
